# Insect herbivory in a mature *Eucalyptus* woodland canopy depends on leaf phenology but not CO_2_ enrichment

**DOI:** 10.1186/s12898-016-0102-z

**Published:** 2016-10-19

**Authors:** Andrew N. Gherlenda, Ben D. Moore, Anthony M. Haigh, Scott N. Johnson, Markus Riegler

**Affiliations:** 1 Hawkesbury Institute for the Environment, Western Sydney University, Locked Bag 1797, Penrith, NSW 2751 Australia; 2 School of Science and Health, Western Sydney University, Locked Bag 1797, Penrith, NSW 2751 Australia

**Keywords:** Arthropod, Climate change, Eucalypt, FACE, Plant–insect interaction

## Abstract

**Background:**

Climate change factors such as elevated atmospheric carbon dioxide concentrations (e[CO_2_]) and altered rainfall patterns can alter leaf composition and phenology. This may subsequently impact insect herbivory. In sclerophyllous forests insects have developed strategies, such as preferentially feeding on new leaf growth, to overcome physical or foliar nitrogen constraints, and this may shift under climate change. Few studies of insect herbivory at elevated [CO_2_] have occurred under field conditions and none on mature evergreen trees in a naturally established forest, yet estimates for leaf area loss due to herbivory are required in order to allow accurate predictions of plant productivity in future climates. Here, we assessed herbivory in the upper canopy of mature *Eucalyptus tereticornis* trees at the nutrient-limited *Eucalyptus* free-air CO_2_ enrichment (EucFACE) experiment during the first 19 months of CO_2_ enrichment. The assessment of herbivory extended over two consecutive spring—summer periods, with a first survey during four months of the [CO_2_] ramp-up phase after which full [CO_2_] operation was maintained, followed by a second survey period from months 13 to 19.

**Results:**

Throughout the first 2 years of EucFACE, young, expanding leaves sustained significantly greater damage from insect herbivory (between 25 and 32 % leaf area loss) compared to old or fully expanded leaves (less than 2 % leaf area loss). This preference of insect herbivores for young expanding leaves combined with discontinuous production of new foliage, which occurred in response to rainfall, resulted in monthly variations in leaf herbivory. In contrast to the significant effects of rainfall-driven leaf phenology, elevated [CO_2_] had no effect on leaf consumption or preference of insect herbivores for different leaf age classes.

**Conclusions:**

In the studied nutrient-limited natural *Eucalyptus* woodland, herbivory contributes to a significant loss of young foliage. Leaf phenology is a significant factor that determines the level of herbivory experienced in this evergreen sclerophyllous woodland system, and may therefore also influence the population dynamics of insect herbivores. Furthermore, leaf phenology appears more strongly impacted by rainfall patterns than by e[CO_2_]. e[CO_2_] responses of herbivores on mature trees may only become apparent after extensive CO_2_ fumigation periods.

**Electronic supplementary material:**

The online version of this article (doi:10.1186/s12898-016-0102-z) contains supplementary material, which is available to authorized users.

## Background

Climate change and its drivers can have a significant impact on the physiology, abundance and distribution of insect herbivores [1–3]. Elevated CO_2_ concentrations (e[CO_2_]) often reduce the growth and survival of insect herbivores as a plant-mediated effect influenced by the decrease in leaf nitrogen concentrations [4–7] and an increase in secondary metabolites, such as phenolic compounds [8–10] generally observed at e[CO_2_]. Furthermore, some studies suggest modulation of plant hormone signalling and induced plant defence at e[CO_2_] [9, 10]. Climate change may also alter the timing and amount of precipitation, and this can potentially impact insect abundance and phenology both directly, and indirectly as a consequence of changes in plant phenology and productivity [11–13]. It has been demonstrated that e[CO_2_] can increase plant net primary production (NPP) [14, 15] and this could potentially benefit insect herbivores as a result of greater resource availability. However, this increase in NPP may be constrained or even reduced in nutrient-limited [16, 17] or water-limited forests [18], or also due to changing herbivory patterns. Furthermore, the measurement of NPP in field experiments may be underestimated if the impacts of herbivory are not measured, in particular the failure of new leaves to expand, due to herbivory on meristems and very young expanding leaves.

*Eucalyptus* (Myrtaceae) is both an ecologically and economically important tree genus in many parts of the world [19, 20]. *Eucalyptus* species are often characterised by sclerophyllous leaves and are often associated with low-fertility soils common in Australia [21–23]. As for all plants, the chemical and physical properties of *Eucalyptus* leaves change with age; young leaves typically have higher nitrogen concentration and moisture content, and reduced toughness compared to older leaves [11, 24, 25]. These factors increase palatability of young foliage to many herbivorous insects, and this can result in enhanced insect performance when feeding on young compared to older leaves [26–28]. Changes in the amount and occurrence of rainfall events may alter the relationship of insect herbivores with leaf phenology, potentially affecting diversity and abundance of insects within these forests. Furthermore, many plants, including *Eucalyptus*, invest heavily in secondary defence compounds [29, 30], and the production of these secondary compounds may vary throughout leaf development [29, 31, 32]. Herbivore induced plant defence, however, does not appear to occur in *Eucalyptus* [33] but see [34].

Increased consumption of leaves, or compensatory feeding, is often observed in herbivorous insects as a response to plants grown under e[CO_2_]—this is to compensate for the dilution of leaf nitrogen [5]. Leaf consumption by herbivorous insects at e[CO_2_] may result in an additional 17–40 % of leaf damage compared to current levels [7, 35]. Despite the potential for compensatory feeding at e[CO_2_], the survival of herbivorous insects may be reduced while developmental time is often increased [5, 36–38]. Therefore, e[CO_2_] may increase leaf damage caused by individual insects to forest trees, however, the abundances of insects in these forests may be reduced due to the negative effects of e[CO_2_] on insect survival and development. Overall this may result in no net change of leaf damage to trees at e[CO_2_].

It has previously been demonstrated that the production of insect herbivore excrements (or frass), a crude proxy for insect abundance and herbivory, increased after large rainfall events during the spring and summer at the *Eucalyptus* free-air CO_2_ enrichment (EucFACE) experiment [11]. This increase in insect activity coincided with an increase in leaf area index (LAI) at EucFACE [18], suggesting a direct link between the abundance of *Eucalyptus*-feeding insects and leaf phenology. However no e[CO_2_] effects on frass deposition or LAI changes within the first 2 years of EucFACE were found, suggesting that insect herbivory and canopy processes may not be impacted by early stages of [CO_2_] fumigation at EucFACE.

The measurement of frass deposition onto the woodland floor does not reveal which leaf age class experiences most damage from herbivory. Furthermore, e[CO_2_] may change leaf phenology and thereby resource availability for herbivores. It may also alter the preference of insect herbivores for different leaf stages if the relative palatability of young expanding versus fully expanded (mature) or old leaves changes under e[CO_2_]. Any change in the consumption of young expanding leaves may therefore affect the recruitment of new leaves in the forest canopy, and place stress on plants. For new leaves, LAI measurement methods may struggle to discriminate between insect removal of leaf area and reductions in NPP. This can result in an incorrect estimate and under-evaluation of NPP of forests, particularly if climate change factors alter the herbivory of new leaf production.

This study investigated the relationship between insect herbivory and leaf phenology of *Eucalyptus tereticornis* Sm., and the impacts of e[CO_2_] and rainfall patterns on these processes in a mature, evergreen canopy of this tree species forming a naturally established woodland at the EucFACE experimental site. We hypothesised that rates of insect herbivory would respond to new leaf production which again would vary across time based on rainfall. It has previously been demonstrated that rainfall is the key driver of *Eucalyptus* leaf phenology, including at the study site [18, 39, 40]. The aims of this study were to: (1) compare the monthly levels of insect leaf herbivory under ambient and e[CO_2_] conditions within a mature *Eucalyptus* canopy forming a woodland for two spring and summer periods at which herbivore activity was observed to be highest in the first and second year of EucFACE [11]; (2) provide estimates of leaf damage for different leaf age classes (young, mature, old) during the same two time periods which included the major new leaf production events of *E. tereticornis* [18], and (3) determine whether specific leaf age classes were preferred by insect herbivores and if this preference was altered under e[CO_2_] during the first 2 years of EucFACE.

## Methods

### Study site

This study was conducted at the *Eucalyptus* free-air CO_2_ enrichment (EucFACE) experiment located within a native Cumberland Plain woodland remnant [41, 42] in Richmond, NSW, Australia (33°37′S, 150°44′E). The vegetation at EucFACE has been undisturbed for at least 75 years and retains old-growth trees mixed with some re-growth. The vegetation community within the study site is characterised as Cumberland Shale Plains Woodland [41], with mature *E. tereticornis* as the only canopy forming tree species. The site has an open canopy, approximately 600 trees ha^−1^ [43], with a low density of forbs and occasional shrubs in the understorey, together with a diverse community of grasses. The site is on a loamy sand soil of the Richmond Formation [44], which is phosphorus-poor and limits tree growth at the site [45]. The average monthly temperature at the site during the time period of this study was 20 °C with an average monthly rainfall of 73 mm (Additional file 1: Fig. S1).

Six large 25 m diameter rings with a height of 28 m above ground, extending above the tree canopy, were constructed amongst the vegetation of the site. Adjacent to each ring stands a high canopy crane with a person basket that allows access to the canopy from above. Each ring also contains a central scaffold tower. Three rings were fumigated diurnally with CO_2_ enriched air via a proportional-integral-derivative control algorithm [46], while the three remaining rings were control rings, fumigated with ambient air. Beginning in September 2012, the target [CO_2_] in treatment rings was increased by 30 µmol mol^−1^ every month until February 2013; thereafter diurnal [CO_2_] targets within the treatment rings were 150 µmol mol^−1^ above ambient levels of ~400 µmol mol^−1^. Rainfall was recorded using automated tipping bucket gauges (Tipping Bucket Rain gauge TB4, Hydrological Services Pty Ltd, Liverpool, NSW, Australia) located 23.5 m above the ground on the central tower in three rings. Data from these sensors were logged every 15 min using CR3000 data loggers (Campbell Scientific, Townsville, Australia).

### Leaf herbivory and leaf production measurement

From each of the six EucFACE rings three trees were randomly selected and marked in the first year, and a different set of three trees per ring was selected and marked in the second year. The upper canopy (approximately 17 m above-ground) was accessed using the canopy cranes to establish herbivory observation points on each selected tree. For each year, 14 branches per tree were tagged. We expected that the herbivory measured in the upper canopy was representative for the entire tree canopy because it had previously been demonstrated that *Eucalyptus* trees display a homogeneous pattern of herbivory throughout the crown [47]. Leaves on each branch were numbered sequentially from the base to the proximal end. A black permanent marker was used to mark the abaxial leaf surface near the petiole, and this has previously been demonstrated not to alter leaf formation or herbivory [48]. New leaves were marked as they emerged behind the shoot tip. Branches and leaves were initially marked in October 2012 and branches were then monitored monthly until February 2013 during the CO_2_ ramp-up phase (year 1). Three different trees per ring were selected, marked and observed monthly in the second monitoring period from August 2013 to March 2014 (year 2).

The surveyed periods coincided with the majority of chewing insect herbivore activity, as measured by frass deposition to the woodland floor [11] and the growth period of *E. tereticornis* during the austral spring and summer as measured by changes in LAI [18]. For each of the 2 years, the initial measurements of leaf area in October 2012 and August 2013 were used as a baseline to measure subsequent leaf consumption. For this purpose, approximately 100 leaves across the 14 branches per tree were marked for the monitoring throughout the consecutive months. Branches were selected for ease of access with crane and away from scaffolding to reduce risk of mechanical leaf damage or loss. Branches were then surveyed once each month for a period of four months (year 1) and seven months (year 2) and assessed for leaf damage that can be attributed to insect herbivores due to feeding marks and new leaf emergence. Leaf damage due to insect herbivores was recorded in two different ways: firstly leaves were classified into three age classes (see below) and monthly leaf damage was measured as damage within each of these age classes following the formula below; secondly cumulative leaf damage was calculated for individual leaves throughout their development during the two observation periods in year 1 and year 2 of EucFACE. Thus, cumulative leaf damage refers to the total amount of damage occurring within each monitoring period and not over the life of a particular leaf.

Based on size, colour, shape and texture, leaves were assigned to one of three age classes for each canopy survey point: young (new expanding leaves), mature (fully expanded) and old leaves [48]. Age class-specific herbivory was then calculated as average damage to leaves of each age class throughout the observation periods of each year. For each survey month, scaled digital photographs were taken of leaves that were still attached to branches. For this purpose, leaves were flattened between a scaled white board and a clear non-reflective plastic sheet [49]. Photographs from each month were then compared to photographs taken in the previous month. For each month, existing leaf area (LA_e_) was quantified using Adobe Photoshop CS5 (Adobe Systems Incorporated, California, USA) by manually tracing the leaf using scaled photographs. Potential leaf area (LA_p_) i.e. the extent of the leaf area if herbivory had not occurred, was determined by manually drawing and digitally reconstructing the leaf [50]. Skeletonising, mining, and leaf rolling were rarely observed on marked leaves, and therefore disregarded in this study. The recorded damage was exclusively due to removal of leaf area by chewing insects. Monthly increments of leaf consumption were determined with the formula [48]:

$$Lc_{(n + 1)} = \left( {1 {-} \left( {\left( {\frac{{LA_{e} }}{{LA_{p} }}} \right) {-} Lc_{n} } \right)} \right)*100$$

where *Lc*_(n+1)_ is the proportion (% missing) of leaf area consumed within the observation period of one month; *LA*_*e*_ is the actual leaf area recorded for that month; *LA*_*p*_ is the potential leaf area if herbivory had not occurred; and *Lc*_*n*_ is the proportion of herbivory that had occurred in the previous month.

Total leaf consumption at the end of each monitoring period in years 1 and 2 was determined by the sum of the monthly leaf consumption for each respective leaf age class per branch per tree for the monitored period. Loss of entire leaves due to herbivory was distinguished from leaf loss that may occur as a consequence of leaf senescence and abscission, or due to wind. A leaf that had completely disappeared was considered as lost due to herbivory if it had signs of herbivory in the previous month, or if an entire leaf that was undamaged in the previous month had disappeared except for its petiole. A leaf without signs of herbivory in the previous month was considered lost due to senescence and abscission, or due to wind, if it had disappeared together with its petiole. In this case no value of herbivory was assigned. This approach to assign complete loss of leaves due to herbivory is a conservative measure as it only covers known herbivory. Leaf production was determined by the average number of young new expanding leaves present per branch divided by the total number of leaves per branch and averaged per tree.

### Statistical analysis

Linear mixed effects models were constructed using *nlme* [51] in R [v3.2.2, 52]. The fixed model contained [CO_2_], month and their interaction. The random model included ring with tree as a nested factor to account for repeated measures. An autocorrelation function was used in order to test for temporal autocorrelation within years, and an autoregressive moving average (ARMA) correlation structure was employed to model dependence among observations of leaf consumption and leaf production across months using a first-order autoregressive structure (AR1) [53]. The number of expanding leaves present was log + 1 transformed to normalise the model-standardised residuals. The relationships of monthly leaf damage with both the average number of young leaves per branch and with rainfall, were modelled using linear mixed effects models and R^2^ values were obtained using the *r.squaredGLMM* function in the *MuMIn* R package [54, 55].

## Results

**Fig. 1:**
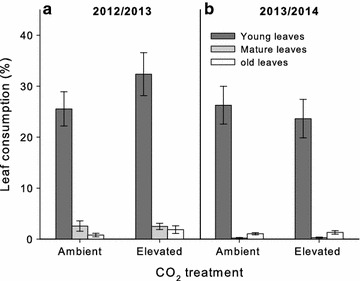
Leaf consumption during three different leaf age classes; young (expanding leaves, *dark grey bars*), mature (fully expanded, *light grey bars*), and old leaves (*open bars*) of mature *Eucalyptus tereticornis* trees grown at ambient or elevated [CO_2_] across two time periods, in year 1 (**a**) and year 2 (**b**). The *figure inset* indicates leaf age

Approximately 3000 *E. tereticornis* leaves were measured for herbivory in each year across three different age classes. Leaf age classes had a significant effect on leaf consumption both in year 1 (*F*_2,30_ = 245.654, *P* < 0.001; Fig. 1a) and in year 2 (*F*_2,30_ = 286.435, *P* < 0.001; Fig. 1b). Young leaves incurred approximately ten times more leaf damage than either mature or old leaves (averages ranged between 25 and 32 % loss in leaf area for young leaves versus less than 2 % loss for mature or old leaves) in both years (Fig. 1). No significant CO_2_ treatment effect on leaf consumption was observed across leaf age classes (year 1: *F*_1,4_ = 0.399, *P* = 0.562; year 2: *F*_1,4_ = 0.042, *P* = 0.848; Fig. 1).

**Fig. 2:**
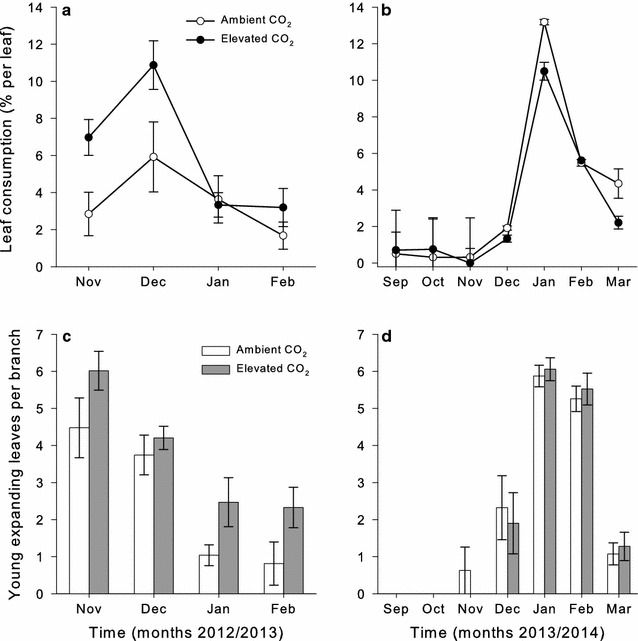
Monthly leaf consumption experienced by all leaf age classes in year 1 (**a**) and year 2 (**b**), and the average number of young expanding leaves observed per branch in year 1 (**c**) and year 2 (**d**) on mature *Eucalyptus tereticornis* trees exposed to ambient (*open circles* or *bars*) or elevated (*closed circles* or *bars*) [CO_2_] at the EucFACE site. The *figure insets* indicate CO_2_ treatment

Significant temporal variation in the amount of leaf consumption during the monitoring periods was observed in both years (year 1: *F*_3,48_ = 10.108, *P* < 0.001; year 2: *F*_6,92_ = 30.998, *P* < 0.001; Fig. 2). Monthly leaf consumption peaked in December in the first year and in January in the second year. No significant difference in monthly leaf consumption was observed between CO_2_ treatments in either year (year 1: *F*_1,4_ = 3.992, *P* = 0.116; year 2: *F*_1,4_ = 0.028, *P* = 0.876; Fig. 2). Total cumulative leaf consumption observed during the monitoring periods did not differ between CO_2_ treatments (year 1: *F*_1,4_ = 6.341, *P* = 0.066; year 2: *F*_1,4_ = 1.681, *P* = 0.265; Table 1). The loss of young leaf production between the two years was nearing significance, with less young leaf production being lost in the second year (*F*_1,4_ = , *P* = 0.051; Table 1).

**Table 1:** Mean percentage (±SE) of total cumulative leaf damage, young leaf production completely lost to herbivory and young leaves which remained undamaged during the expansion stage on mature *E. tereticornis* under ambient or elevated [CO_2_] at the EucFACE site over 2 years

CO_2_ treatment	Year 1		Year 2	
	Ambient	Elevated	Ambient	Elevated
Total cumulative leaf consumption (%)	9.9 ± 1.7	16.6 ± 1.1	13.5 ± 2.5	9.0 ± 0.7
Young leaf production completely lost to herbivory (%)	21.0 ± 5.6	17.3 ± 3.5	9.7 ± 4.3	8.0 ± 3.7
Young leaf production remaining undamaged (%)	39.3 ± 5.9	27.5 ± 3.5	43.7 ± 3.7	38.0 ± 4.2
Young leaf production damaged (%)	39.7 ± 4.4	55.2 ± 3.6	48.7 ± 3.8	54.0 ± 4.4

The value of young leaf production that was damaged is complementary to the lost and undamaged new leaf production values

**Fig. 3:**
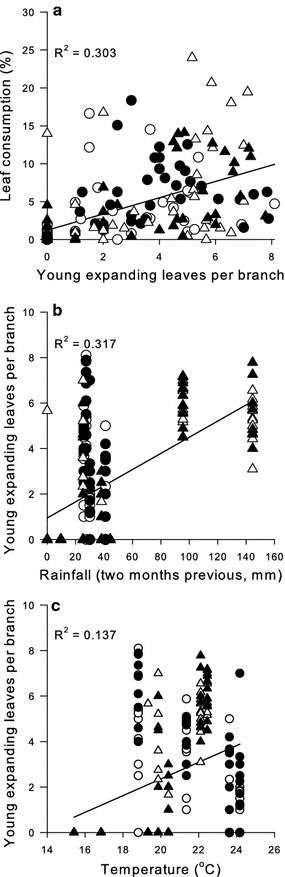
Linear mixed effect model regression of young leaf production per branch and monthly leaf consumption (**a**), rainfall from two months prior and leaf production per branch (**b**), and temperature and leaf production per branch (**c**) observed on *Eucalyptus tereticornis* trees at the EucFACE site under ambient (*open symbols*) or elevated (*filled symbols*) [CO_2_] in year 1 (*circles*) and year 2 (*triangles*)

The average number of young leaves present per branch in the first year peaked in November (*F*_3,48_ = 21.999, *P* < 0.001; Fig. 2c) while production of young leaves was highest in January and February of year 2 (*F*_6,92_ = 68.570, *P* < 0.001; Fig. 2d). No significant differences in young expanding leaf production were observed between CO_2_ treatments in either year (year 1: *F*_1,4_ = 7.075, *P* = 0.056; year 2 *F*_1,4_ = 0.114, *P* = 0.753). Furthermore, e[CO_2_] did not alter the timing of flush production between year 1 and 2 (*F*_1,167_ = 3.004, *P* = 0.085). Strong positive correlations were observed between the number of expanding leaves present and leaf consumption (R^2^ = 0.303, d.f. = 161, *P* < 0.001; Fig. 3a). Rainfall that occurred two months prior to the monitoring data points was positively correlated with leaf production (R^2^ = 0.317, d.f. = 161, *P* < 0.001; Fig. 3b). Temperature was also positively correlated with leaf production (R^2^ = 0.137, d.f. = 161, *P* < 0.001; Fig. 3c).

The number of new young leaves produced by trees and lost to herbivory did not differ as a result of CO_2_ treatment in either year (year 1: *F*_1,4_ = 0.385, *P* = 0.569; year 2: *F*_1,4_ = 0.148, *P* = 0.720; Table 1). Overall, approximately 37 % of new young leaves produced by trees escaped any form of chewing damage (Table 1). CO_2_ treatments did not affect this percentage of young leaves escaping any form of chewing damage in either years (year 1: *F*_1,4_ = 0.043, *P* = 0.846; year 2: *F*_1,4_ = 1.060, *P* = 0.361).

## Discussion

Herbivory by chewing insects was measured over the first 2 years of the EucFACE experiment. In the first year, e[CO_2_] was gradually increased from ambient conditions to +150 µmol mol^−1^, while in the second year, e[CO_2_] was maintained at 550 µmol mol^−1^. The two monitoring periods included two major leaf production events, one in each year during spring and summer. Independent of [CO_2_], consumption of young expanding leaves was very high and decreased to very low levels once leaves were fully expanded. The drastically higher levels of herbivory on young leaves drove monthly variations in overall leaf damage by insect herbivores due to the variation in leaf production. Furthermore, e[CO_2_] did not affect damage due to herbivory and total leaf consumption. This was in line with our expectation that we would not detect compensatory feeding because of a previous study in which we detected that concentrations of foliar nitrogen and total phenolics at the EucFACE site were not affected by CO_2_ fumigation over the first 2 years of EucFACE [11]. Such a lack in plant responses may be due to the capacity of mature trees to compensate short term e[CO_2_] exposure by retrieving nutrient reserves [11].

We found that herbivory by leaf chewing insects increased with the production of new young leaves suggesting that insect feeding is synchronised with the emergence of new young leaves. Insects generally prefer young expanding leaves over mature and old leaves as a result of higher nutrient content and lower physical defences in young leaves, in particular in sclerophyllous *Eucalyptus* leaves [27, 36, 48, 56]. We have previously demonstrated that foliar nitrogen concentration was higher in flush growth than mature leaves at EucFACE, independent of [CO_2_] [11]. Herbivory decreased to very low levels in fully expanded leaves, suggesting that leaf flush chewers rather than senescent leaf chewers were the dominant feeding guild during the monitoring periods of this study. Frass production over the same time period [11] displayed a similar pattern of increased deposition around the periods of new leaf production [18]. This highlights the importance of new leaf production not only to insect herbivores but also for insect-mediated nutrient cycling within forests [11].

Herbivory on *E. tereticornis* in a native woodland observed in this study is within the range observed in other Australian systems. For example, in an Australian rainforest insect herbivore damage on young expanding leaves ranged between 10 and 30 % depending on tree species, and this fell to less than 5 % once leaves matured [48]. Similarly, Moles and Westoby [57] reported that leaf damage to expanding leaves from 51 woody dicotyledonous species in a coastal dry sclerophyll forest ranged between 0 and 51 %. Irrespective of leaf age, reported total leaf herbivore damage in Australian forests ranges between 5 and 44 % [23, 47, 58, 59]. Overall, at EucFACE we observed total herbivore consumption of between 9 and 17 % of leaf area, which is in the lower range of previous studies conducted in Australian forests. No leaf mining or leaf rolling was observed; therefore the damage measured in this study was exclusively due to leaf chewing insects. This is often the most common type of leaf damage observed in forests, when compared to other insect feeding types [59–61]. The feeding guild of leaf chewers is also the most likely impacted by e[CO_2_] [7]. A previous study of herbivory on *E. tereticornis* at the study site also identified leaf chewers as the dominant feeding guild [62].

The responses to eCO_2_ of two key leaf chewing insect herbivores found at the EucFACE site had previously been tested on young *E. tereticornis* trees in greenhouse experiments. The cup moth *Doratifera quadriguttata* (Lepidoptera: Limacodidae) and the leaf beetle *Paropsis atomaria* (Coleoptera: Chrysomelidae), both experienced negative effects of e[CO_2_] such as increased mortality, extended developmental times and reduced pupal weights, while both species also displayed signs of compensatory feeding [36–38]. However, we did not observe any differences in the amount of leaf damage occurring to mature *E. tereticornis* trees at EucFACE under CO_2_ enrichment. In other forests undergoing CO_2_ fumigation e[CO_2_] has been observed to either decrease [63, 64], cause no change [65], or increase [66, 67] the amount of leaf damage. These differences in herbivory responses to e[CO_2_] across a variety of forest types may be due to the complexity in interactions between biotic and abiotic factors which impact insect herbivores and therefore herbivory.

The different timing of the major flush production events between the 2 years at EucFACE may have affected the composition of herbivore communities within the woodland. Shifts in the timing of flush growth may have detrimental effects on herbivores that depend on flush with the potential outcome of an altered insect herbivore community structure. Although we did not directly assess insect populations our data indirectly suggests that such potential changes in chewing insect herbivore populations may have occurred. It appeared that less of the newly produced leaves were consumed in the second year, potentially due to the altered timing of flush growth. This may also indicate that there was a shift away from flush leaf chewers. In another study that assessed deposition of lerp (small covers produced by plant sap-feeding psyllids) to the woodland floor, a rapid outbreak succession by two psyllid species, *Glycaspis* sp. and *Cardiaspina fiscella* was detected on *E. tereticornis* after March 2014 [68]. This suggests that a rapid change in the overall canopy insect community composition must have occurred directly after the leaf phenology and herbivory surveys presented here. Our previous study on frass deposition by leaf chewing insects also demonstrated that less frass was produced in 2013/2014 (year 2) than in 2012/2013 (year 1) [11].

Our leaf herbivory study only focussed on leaf-chewing insects, and sap-feeding insects were not considered here. This focus on the leaf-chewing feeding guild may underestimate the impacts of herbivory occurring in the canopy and the true level of canopy biomass lost to insect herbivores in our study site. Significant numbers of plant sap-feeding psyllids were detected at EucFACE after March 2014 [68]. Some psyllid species can be significant leaf defoliators of *Eucalyptus*. For example, *Cardiaspina* sp. has caused area-wide defoliation on *Eucalyptus moluccana* but not any other *Eucalyptus* species in the Cumberland Plain Woodlands since 2009 [69]. Similarly, *C. fiscella* caused significant defoliation of the EucFACE site at the end of 2014 [68], after the end of the study presented here. This defoliation occurred because *Cardiaspina* can induce leaf senescence and defoliation [34]. However, throughout the surveying period of our herbivory study at EucFACE (from October 2012 until March 2014) we did not observe any high abundance of leaf defoliating psyllids [68]. After the ramp-up phase (from March 2013 to December 2014) the abundance of lerps deposited to the woodland floor was reduced for three psyllid species at e[CO_2_], and signs for compensatory feeding were detected in one of these three species, *Glycaspis* sp. [68]. These differences were recorded despite the absence of any measureable differences in N concentration in leaves [11].

The loss of young expanding leaves, as found in our study, may be more detrimental to plants than the loss of mature leaves, because the energy and resources invested in the production of new leaves have yet to be recovered [48]. This may impact forest growth and the estimation of carbon storage potential within forests and highlights the need to consider the role of herbivorous insects in ecosystem functioning [66]. Removal of leaf material by insect herbivores is often not accounted for in models of plant productivity as common techniques used to measure productivity, such as LAI [70, 71], fail to account for the loss of newly produced plant material to insect herbivory. This can complicate estimates of NPP and may underestimate true productivity of forests and utilisation of forest resources. Furthermore, plant NPP often increases at e[CO_2_] as a result of a carbon fertilisation effect [72, 73]. However, in the early stages of CO_2_ fumigation at the EucFACE site we did not observe an increase in leaf production. This may indicate that mature *E. tereticornis* trees within the site are limited by water and nutrient availability [18, 45], and increased photosynthesis due to e[CO_2_] may also result in increased respiration in the ecosystem [43] rather than the production of more leaves.

## Conclusions

Predictions about damage inflicted by insect herbivores at e[CO_2_] are difficult to make under field conditions owing to the complex interactions between plants, insect herbivores and their antagonists. This uncertainty in insect herbivore responses hinders the ability to accurately determine model-specifications of their response to climate change [74]. Contrary to our original expectations, CO_2_ fumigation at the EucFACE site for the first 2 years did not affect total leaf consumption by herbivores or their leaf age preference, in part likely due to the lack of e[CO_2_] responses in concentrations of foliar nitrogen and total phenolics in insect frass [11]. However, it is clear that new and expanding leaves were heavily damaged while fully expanded leaves were not. Damage on young foliage is often not accounted for in estimates of forest productivity, yet this can amount to substantial underestimates of true forest productivity. Rainfall-mediated production of new leaves is an important regulator of insect herbivory in sclerophyllous forests due to the physical barriers to consumption present in mature leaves [75], and this will require further attention in climate change studies. Shifts in rainfall patterns, a potential outcome of climate change [76], can have significant effects on insect community composition, herbivory and insect frass deposition. This may have detrimental or positive outcomes for ecosystems and humans, for example by stimulating pest populations, or regulating ecosystem services that insects provide in forests and managed plantations. Understanding how rainfall may interact with e[CO_2_] in altering insect herbivory and herbivore abundances is important in predicting the impacts of climate change variables on insect herbivore population dynamics in forests and plantations.

## Additional file

**Additional file 1: Fig. S1.** Total monthly rainfall (*bars*) and average monthly temperature (*circles*) at the EucFACE site.

## Abbreviations

e[CO_2_]: elevated carbon dioxide concentrations
EucFACE: *Eucalyptus* free-air CO_2_ enrichment
LA_e_: existing leaf area
LAI: leaf area index
LA_p_: potential leaf area
*Lc*_*n*_: proportion of herbivory that had occurred in the previous month
NPP: net primary production
