# Supplementary material for: Insect herbivory in a mature Eucalyptus woodland canopy depends on leaf phenology but not CO2 enrichment
Source: BMC Ecol. 2016 Oct 19;16:47. doi: 10.1186/s12898-016-0102-z (PMC5072302; doi:10.1186/s12898-016-0102-z)
Supplement: Supplementary file 1 — Additional file 1: Fig. S1. Total monthly rainfall (bars) and average monthly temperature (circles) at the EucFACE site. [file 12898_2016_102_MOESM1_ESM.docx]

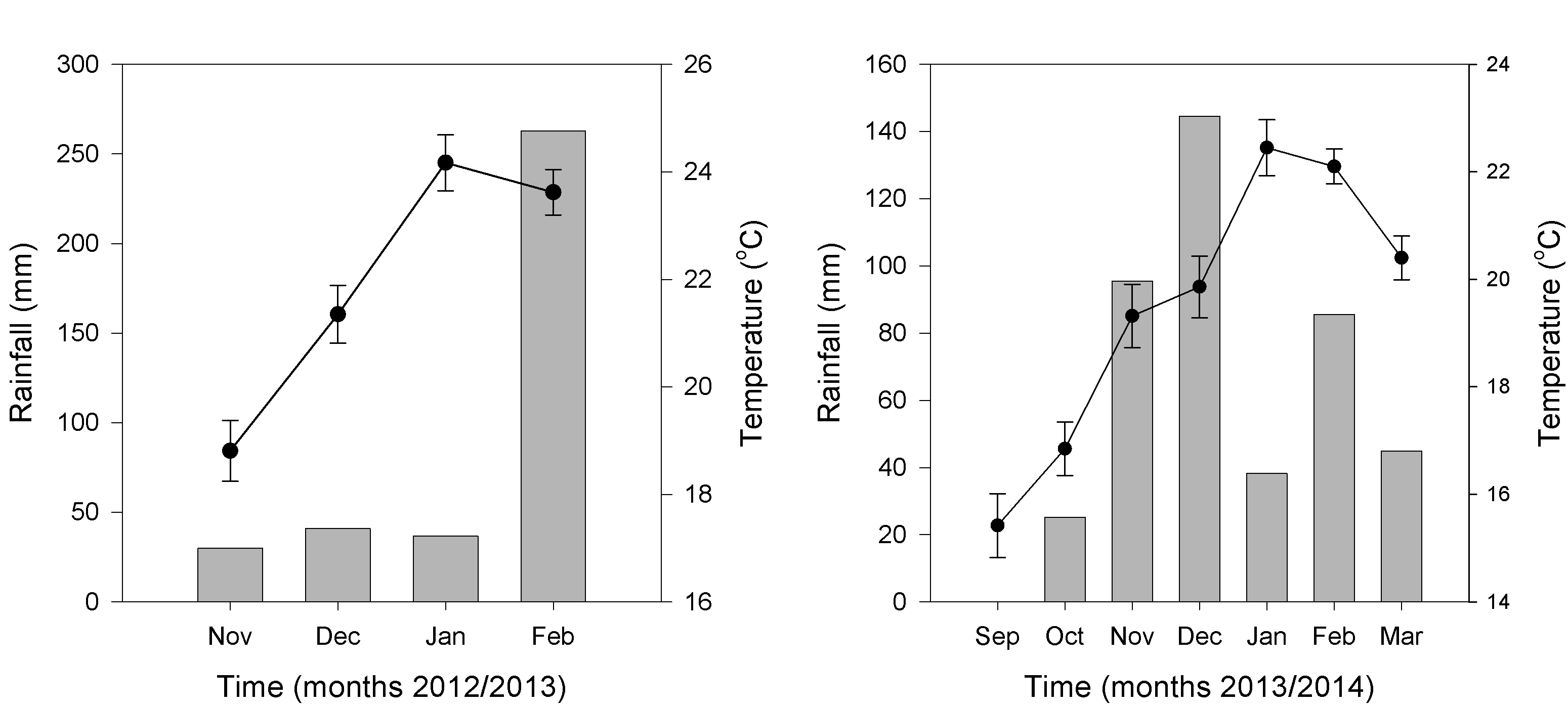


Figure S1. Total monthly rainfall (bars) and average monthly temperature (circles) at the EucFACE site.
